# Reciprocal Cooperation of Type A Procyanidin and Nitrofurantoin Against Multi-Drug Resistant (MDR) UPEC: A pH-Dependent Study

**DOI:** 10.3389/fcimb.2020.00421

**Published:** 2020-08-11

**Authors:** Sahana Vasudevan, Gopalakrishnan Thamil Selvan, Sunil Bhaskaran, Natarajan Hari, Adline Princy Solomon

**Affiliations:** ^1^Quorum Sensing Laboratory, Centre for Research in Infectious Diseases (CRID), School of Chemical and Biotechnology, SASTRA Deemed to be University, Thanjavur, India; ^2^Department of Scientific Affairs, Indus Biotech Private Limited, Pune, India; ^3^Nuclear Magnetic Resonance Laboratory, School of Chemical and Biotechnology, SASTRA Deemed to be University, Thanjavur, India

**Keywords:** UPEC, type A procyanidin, nitrofurantoin, anti-biofilm, adhesins

## Abstract

Uropathogenic *Escherichia coli* (UPEC) accounts for the majority of complicated and uncomplicated urinary tract infections. The use of phytomolecules in the treatment of UTI is fast gaining attention. The current report identifies a multidrug-resistant strain (QSLUPEC7), which is a strong biofilm producer, among the considered clinical isolates. The antimicrobial and antibiofilm activity was evaluated for the phytomolecule, Type A procyanidin (TAP) from *Cinnamomum zeylanicum* against QSLUPEC7. TAP treatment did not affect the growth of the MDR strain but affected the biofilm formation (~70% inhibition). The confocal microscopic examination reveals the biofilm inhibition and the live cells in the biofilm corroborates the antimicrobial results. Further, the synergy studies of TAP and nitrofurantoin (NIT) were carried out at different pH. TAP acts synergistically with nitrofurantoin at different pH considered. A closer look in the results reveals that at pH 5.8, maximum growth inhibition is recorded. The gene expression analysis shows that TAP alone and in combination with NIT downregulates the major fimbriae adhesins of UPEC. The results conclude that the TAP has an antibiofilm activity against the multidrug-resistant strain of UPEC, without affecting the growth. Also, TAP reciprocally cooperates with nitrofurantoin at different pH by downregulating the adhesins of UPEC.

## Introduction

Urinary tract infections (UTI) are the collective term for pathogenic infections of the urinary tract, which are estimated to cost $5 billion annually (Tan and Chlebicki, 2016). The opportunistic intracellular pathogen, Uropathogenic *Escherichia coli* (UPEC) accounts for the 80–85% of cases of UTIs. The armament of virulence factors, both structural and secreted, of UPEC directs the adhesion and invasion of UPEC to epithelial cells. These virulence factors, along with the biofilm formation, facilitates UPEC growth, persistence in extreme pH variation, and toxin secretion (Flores-Mireles et al., 2015). In addition to virulence factors, host factors such as urine pH and iron availability in the bladder also influence UPEC behavior (Nielubowicz and Mobley, 2010). The therapeutic response of the current treatments is affected by both high urinary concentrations and urinary pH (Cunha, 2016). pH affects not only the growth of the uropathogens but also the efficacy of antibiotics (Burian et al., 2012). Therefore, it is crucial to take into account the pH of the urine before any treatments. The effect of urinary pH in the antimicrobial action of nitrofurantoin is well-documented (Fransen et al., 2017). Nitrofurantoin is a broad-spectrum antibiotic, exclusively used as a therapy for uncomplicated UTI–cystitis (Gardiner et al., 2019) and a prophylactic agent for recurrent UTI (Muller et al., 2017). It is from the Nitrofurans family of flavonoids and works best at an optimum pH of 5.5–6.5 against UPEC (Fransen et al., 2017). A strategy to improve the efficacy of antibiotics in the face of antibiotic resistance and changing pH is to use combinations of plant-derived compounds with antibiotics, which enhances and restores the antibacterial activity of the traditional antibiotics. This improves antibiotic efficiency as well as reduces the concentration drastically without any gain of resistance (Stapleton et al., 2004; Coutinho et al., 2009; Li, 2016). Plant extracts such as polyphenols are known to cause cell wall lysis and inhibit efflux pumps (Chusri et al., 2009). Cinnamon bark (*Cinnamomum zeylanicum*) is traditionally known for possessing potent biological activities such as antibacterial, antitermitic, larvicidal, antifungal, insecticidal, and nematicidal activities (Nabavi et al., 2015). Cinnamons are known to possess oligomeric procyanidins which confer different biological properties (Rauf et al., 2019). Previous reports show the procyanidins to affect dental caries and suppression of various virulence factors from sorghum episperm (Xu et al., 2011). Type A procyanidin (TAP) extracted from cinnamon was previously shown to improve immune responses and antiviral activity (Bhaskaran and Vishwaraman, 2014). The current study explores the kinetics of synergistic action of the TAP and nitrofurantoin at different pH against MDR UPEC.

## Materials and Methods

### Chemicals and Reagents Used

The cinnamon derived type A procyanidin (TAP) was obtained from Indus Biotech Pvt. Ltd., Pune, India. Nitrofurantoin (NIT) antibiotic was purchased from Sigma Aldrich, USA (98.0–102.0% purity). The antibiotic discs were purchased from HiMedia. The stock concentration (1 mg/mL) of TAP was prepared in sterile distilled water and the stock concentration (50 mg/mL) of nitrofurantoin was prepared in DMSO according to CLSI guidelines. The concentration of DMSO was maintained at >0.5% for all assays.

### Microbial Strains and Conditions

A total of 13 UPEC clinical isolates (QSLUPEC1–QSLUPEC13) were obtained from the microbial repository of JSS medical college, Mysore. These strains were collected at different time points from the patients reported to have urinary tract infections. The isolates were confirmed to be *E. coli* by standard microbiological screening methods. They were also checked for their expression of the *fimX* gene, which is type 1 pili regulator of *E. coli* (Bateman et al., 2013). The strains were maintained as glycerol stock at −80°C. Cation adjusted Muller Hilton Broth (CAMHB) was used for the determination of the antibacterial activity and synergy studies. The biofilm formation and inhibitory effect was done in the Luria Broth media (LB).

### Screening of Multidrug Resistant Strains

To screen the MDR strains, the clinical isolates were screened for resistance to the antibiotics. The following nine antibiotics which belong to different classes were tested for resistance: Co-Trimoxazole (COT), Trimethoprim (TMP), Ampicillin (AMP), Nalidixic acid (NAL), Streptomycin (STS), Cefuroxime (CXM), Cefotaxime (CTX), Norfloxacin (NOR), and Ciprofloxacin (CIP).

A standard disc diffusion assay was carried out for screening the resistant strains. Briefly, the bacterial suspensions were prepared from 16 h old plate. The turbidity was set equivalent to 0.5 McFarland standard (OD_595_ = 0.08–0.1). The OD adjusted bacterial suspensions were swabbed onto Mueller-Hinton agar plates, incubated for 24 h at 37°C. The zone of inhibition was measured and interpreted with CLSI guidelines [CLSI M100–ED30: (Clinical and Laboratory Standards Institute (CLSI), 2020)]. According to the standard definition by Magiorakos et al. (2012), the strains resistant to three and more than three antimicrobial classes were defined as MDR.

### Biofilm Formation Assay

The biofilm-forming capability of the 13 clinical isolates was assessed semi-quantitatively using the standard Tissue Culture Plate (TCP) method and qualitatively using Congo Red Agar (CRA) method (Hassan et al., 2011). For the tissue culture plate method, the overnight plate cultures of each of the clinical isolates were adjusted to 0.5 McFarland units (~1.5 × 10^8^ CFU/mL) with saline media. The prepared suspensions were added to 96 well microtiter plates containing LB media (1:10 dilution). After 24 h incubation at 37°C, absorbance was measured at OD_655_ using ELISA plate reader (BioRad i-Mark, Japan). Then, the planktonic cells were removed by gently tapping the plate and subsequent washing with water twice. The wells were stained with 100 μL of 0.2% crystal violet stain and incubated for 20 min. After air-drying the plate for 30 min, the bound crystal violet was suspended in 33% acetic acid. The optical density was measured at the wavelength of 595 nm. The specific biofilm formation index was calculated as follows:

$$\begin{array}{l} SBF=\;\frac{AB-CW}{G} \end{array}$$

where SBF denotes Specific Biofilm formation index, AB denotes OD_595_ of the stained attached bacteria, CW denotes OD_595_ of the well containing LB media (blank), and G denotes OD_655_ of the cell growth in the suspended culture (Naves et al., 2008).

Qualitatively, biofilm formation was assessed using Congo Red Agar (CRA) method. The congo red agar medium was prepared as described previously (Hassan et al., 2011). The bacterial suspensions were streaked in the CRA media and incubated for 24 h at 37°C. The colonies were visualized, and the strains with black colonies and dry consistency were interpreted as biofilm producers.

### Biofilm Inhibitory Activity of TAP

From the above assays, the strain that has the dual strength of being a strong biofilm producer and multidrug-resistant was chosen to understand the biological activity of TAP.

The biofilm inhibitory efficacy of the TAP was tested at different concentrations (128–2 μg/ml). The bacterial cultures inoculated in the above-mentioned conditions were incubated at 37°C for 24 h without agitation. The bacterial inoculated broth was taken as control and the uninoculated broth was taken as blank. The biofilm was processed using the crystal violet assay. Briefly, the planktonic cells were removed, and the adhered cells were fixed with methanol. The adhered biofilm cells were incubated with 0.2% crystal violet for 30 min at room temperature. The excess stain was washed, and biofilm stained crystal violet was eluted using 33% acetic acid. The optical density was measured at the wavelength of 595 nm. The treated biofilm at different concentrations formed is compared to the untreated culture. The minimum biofilm inhibitory concentration –MBIC_50_ and MBIC_90_ are the lowest concentrations at which the compound inhibits 50 and 90% biofilm as compared to the untreated control, respectively.

### Microscopic Analysis of Biofilm Inhibition

Confocal microscopy imaging was used to examine the biofilm inhibition of TAP. The bacterial suspensions were prepared as described above. The biofilm was allowed to form in clean, sterile coverslips in the presence and absence of the above-mentioned treatment. After 24 h incubation, the planktonic cells from coverslips were removed by rinsing with sterile water and stained with *Bac*Light Bacterial Viability Kit (L7012) as per the kit protocol. A 40X objective lens was used to capture two and three-dimensional images using a confocal laser scanning microscope (Olympus FLUOVIEW, FV1000).

### Checker Board Analysis

The double-dose response of TAP and nitrofurantoin was determined using checkerboard analysis. The overnight plate culture of the MDR strain was adjusted to 0.5 McFarland units (~1.5 × 10^8^ CFU/mL) with saline media. The prepared suspensions were added to 96 well microtiter plates containing CAMHB media (1:10 dilution). The different dilutions were prepared by dissolving TAP in the bacterial growth media (CAMHB). The concentration of TAP and nitrofurantoin for synergy studies were from 64 to 1 μg/ml: 2-fold dilution. The plates were incubated at 37°C for 24 h. After the incubation, the growth inhibition was measured by comparing the OD_595_ of control and the plant polyphenols treated cells.

$$\begin{array}{l} \%\;Growth\;Inhibition=\frac{Control\;OD_{595}-Test\;OD_{595}}{Control\;OD_{595}\;}\;\times 100 \end{array}$$

The synergism was evaluated using the BI (Bliss independence model) and FIC (Fractional Inhibitory Concentration) index. The calculations are similar to the previous reports (Kaur et al., 2016).

### Time-Course Action of the Combination

The synergism kinetics at different pH (5.2, 5.8, 6.4, 7.0, and 7.6) was performed in broth microdilution method for nitrofurantoin and TAP using 96 well microtiter plate. The bacterial suspension was prepared as mentioned above and was added (1:10 dilution) to the CAMHB media containing different concentrations of the TAP and nitrofurantoin (64–1 μg/ml: 2-fold dilution) at varying pH. The plates were incubated at 37°C and absorbance was measured at 595 nm at a time interval of 1–8 h and also after 24 h.

### Gene Expression Analysis

The biofilm inhibitory effect of the synergistic combination was understood using the gene expression analysis of the biofilm regulatory genes of UPEC. The planktonic cells (log phase) of UPEC were treated with Nitrofurantoin (8 μg/mL), TAP (32 μg/mL), a combination of TAP (32 μg/mL) and Nitrofurantoin (8 μg/mL) and incubated for 24 h at 37°C. The pH of 5.8 was maintained in all the treatments. Total RNA was extracted by following the manufacturer's guidelines of RNeasy® Protect Bacteria Mini Kit (Qiagen). Standard agarose gel electrophoresis procedure was developed to verify the integrity and NanoDrop (Thermo Scientific, USA) was done to evaluate the purity of isolated RNA. From the isolated RNA, cDNA was synthesized using iScript™ cDNA Synthesis Kit (Manufacturer's protocol was followed).

The expression level of genes responsible for adhesins of UPEC was analyzed using qRT-PCR. The respective primers and the melting temperature of each of the gene used were listed in Table S1. Reference gene and negative control are 16srRNA and without cDNA were maintained, respectively. Calculations of relative gene expression were done with 2^−ΔΔCT^ method (Hema et al., 2017).

### Statistical Analysis

GraphPad Prism software version 8.0.2 (GraphPad Software Inc., San Diego, CA, United States) was used for carrying out the statistical analysis. The significance was checked with Student *t*-test with *p* set at *p* ≤ 0.05. All the assays were carried out in biological and technical triplicates, and the results were expressed as mean ± SD.

## Results

### Antibiogram of the Clinical Isolates

For the clinical isolates tested, the most common resistance was observed in nalidixic acid, ciprofloxacin and ampicillin, followed by cefuroxime, cefotaxime and norfloxacin, tetracycline, co-trimoxazole, trimethoprim, and streptomycin.

It is interesting to note that no isolate was resistant to all nine antibiotics considered. But all were resistant to at least three antibiotics. Thus, all the strains were multidrug-resistant according to the definition by Magiorakos et al. (2012), QSLUPEC1 and QSLUPEC6 were resistant to at least one agent in three antimicrobial classes whereas the majority of the strains, QSLUPEC2, QSLUPEC9, QSLUPEC10, QSLUPEC11, QSLUPEC12, and QSLUPEC13 were resistant to in four antimicrobial classes. The clinical isolates, QSLUPEC3, QSLUPEC5, QSLUPEC7, and QSLUPEC8 were resistant to at least one agent in five antibiotic categories (Table S2).

### Biofilm Forming Capacity

The biofilm-forming capacity of the clinical isolates was analyzed using TCP method and CRA method. The data are summarized in Table S3. According to Naves et al. (2008), the strains having SBF > 1.10 is considered to be strong biofilm producers, 0.7–1.09 are moderate biofilm producers, 0.35–0.69 are weak biofilm producers. The SBF <0.35 are not capable of biofilm formation.

Of the 13 clinical isolates, seven isolates were strong biofilm producers, five were moderate biofilm producers, and only one was a weak biofilm producer. The CRA method correlated with the TCP method except for one clinical isolate. For QSLUPEC2, which showed a moderate biofilm production in the TCP method and observed pink colonies in the case of CRA method.

The correlation between biofilm formation and antibiotic resistance were next analyzed. Out of the three classified MDR isolates, three were strong biofilm formers (QSLUPEC3, QSLUPEC5, and QSLUPEC7). QSLUPEC8 was a moderate biofilm producer. In order to validate the biofilm inhibition and synergy activity of TAP, QSLUPEC7, which is multidrug-resistant and also a strong biofilm producer, was chosen.

### Biofilm Impairment by TAP

**Figure 1:**
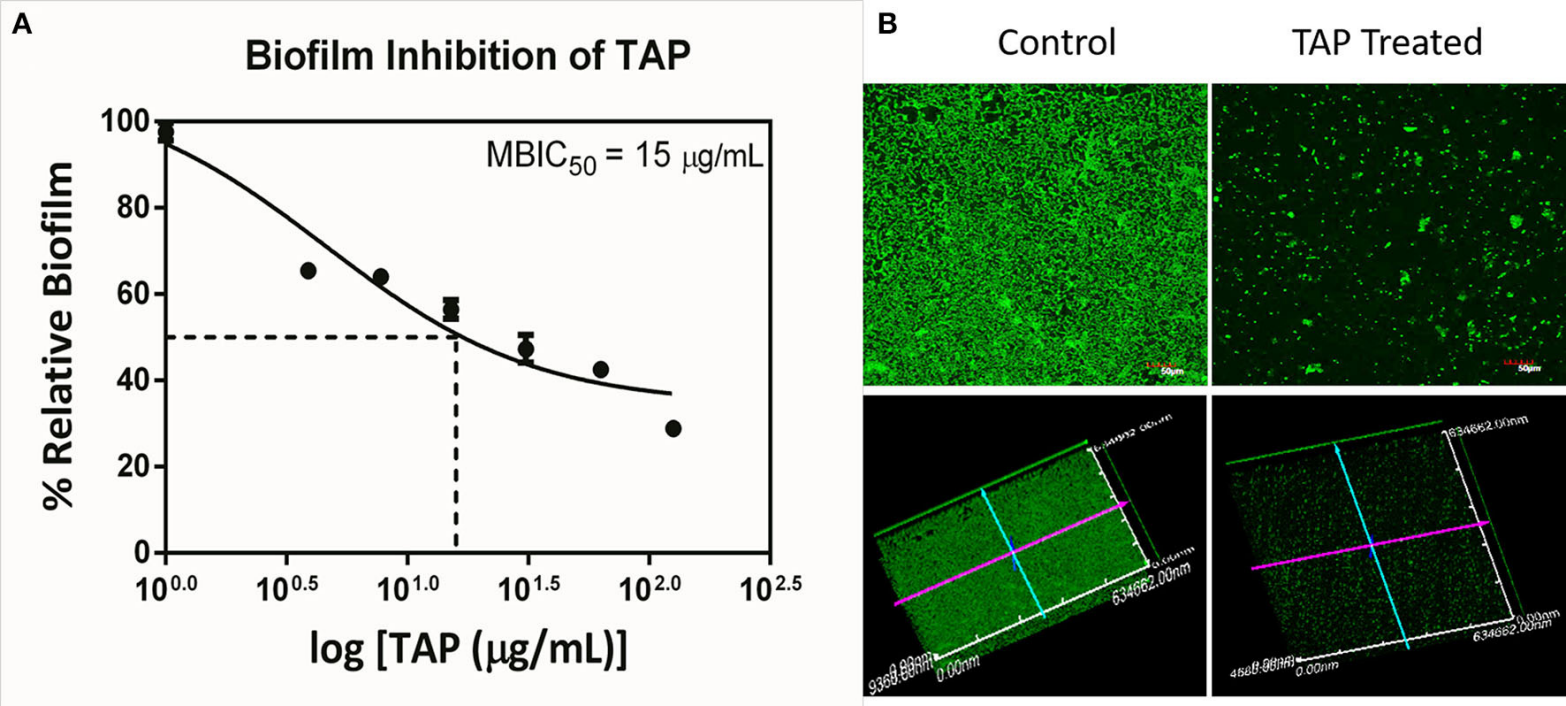
Biofilm Inhibition of TAP. **(A)** The monotonic dose-response curve of biofilm inhibitory action of TAP. The maximum biofilm inhibition was observed at 128 μg/mL. **(B)** The confocal micrographs of the biofilm inhibition at 128 μg/mL. Z-axis length is 10 μm.

The antimicrobial and antibiofilm activity of TAP against the QSLUPEC7 was evaluated. TAP did not have any antimicrobial activity across the concentrations considered (Data not shown). The maximum biofilm inhibitory activity was recorded at 128 μg/mL, and a monotonic dose curve was observed (Figure 1A). The confocal micrographs also substantiated the quantitative results. There was a significant reduction in the biofilm formation with TAP treatment (~71%). The absence of the red fluorescence supported the fact that there was no cell death with the TAP treatment even at the highest concentration considered (Figure 1B).

### Potentiation of Nitrofurantoin by TAP—pH-Dependent Study

**Figure 2:**
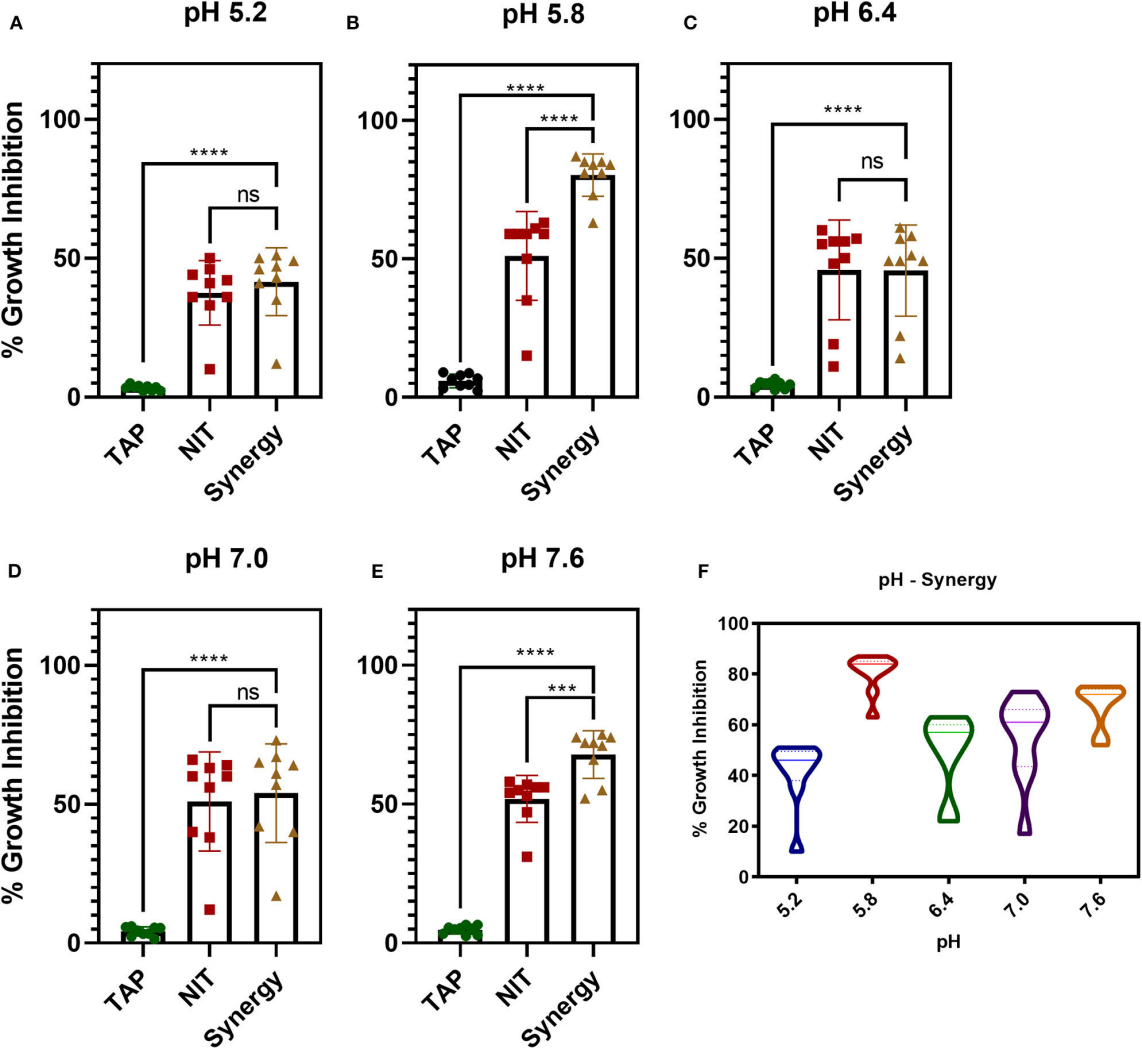
pH and time-dependent synergy activity of TAP and NIT. **(A–E)** shows the individual and synergy antibacterial action at the recorded concentrations of synergy activity. **(A,B)** the concentrations of TAP and NIT are 32 and 8 μg/mL, respectively. **(C–E)** the concentrations of TAP and NIT are 64 and 16 μg/mL, respectively. Student unpaired *t*-test was used for the significance analysis. *p* < 0.05 was considered significant. ***p = 0.002 and ****p ≤ 0.0001. **(F)** Shows the violin plot to depict the distribution of the synergy activity at different time points. At pH 5.8, the activity is maintained across all time points.

The antibiofilm activity at a very low concentration of TAP directed toward the evaluation of potentiating activity of TAP with the known frontline antibiotic, NIT. Previous reports state that the effective pH of nitrofurantoin activity is 5.5–6.5 (Fransen et al., 2017). Thus, the synergy activity was tested in different pH (5.2, 5.8, 6.4, 7.0, and 7.6) in a time-dependent manner. Figure 2 shows the time-dependent antibacterial activity at a different pH and synergy concentration. It is observed that the antimicrobial action increases in a time-dependent manner. Thus, TAP potentiates the activity of nitrofurantoin significantly at pH 5.8 and pH 7.6, as compared to the other pH values considered. Table 1 shows the synergy scores calculated using the BLISS independence model in a pH and time-dependent manner. The Bliss score and FIC index values indicate that synergistic action of the TAP and nitrofurantoin. Taking both the synergy models into the account, a strong synergism was observed for pH 5.8, at a lower concentration of 8 μg/mL of NIT and 32 μg/mL. Even though the FIC index indicated synergy in the case of pH 5.2, the BLISS calculation indicated weak synergy. A moderate synergy was observed for pH 6.4 and pH 7.0. A stronger synergism was observed at pH 7.6 at a concentration of 16 μg/mL of NIT and 64 μg/mL.

**Table 1:** BLISS and FIC calculations at different pH.

**Sl. No**.	**pH**	**Conc. of NIT (μg/mL)**	**Conc. of TAP (μg/mL)**	**Time Point (h)**	**BLISS Score**	**Interpretation**	**FIC Index**	**Interpretation**
1	5.2	8	32	8	62.7	Weak synergy	0.03125	Synergy
				24	41.25	Weak synergy		
2	5.8	8	32	8	377.4	Strong synergy	0.03125	Synergy
				24	418.1	Strong synergy		
3	6.4	32	64	8	120.5	Moderate synergy	0.25	Synergy
				24	154	Moderate synergy		
4	7.0	16	64	8	245	Strong synergy	0.125	Synergy
				24	186.6	Moderate synergy		
5	7.6	16	64	8	153	Moderate synergy	0.125	Synergy
				24	208	Strong synergy		

### Synergy Activity—Time Course Action

Figure 2E shows a violin plot of the synergy activity at different pH considered in a time-dependent fashion. The maximum inhibition of 43% was recorded for pH 5.2 at 8 h and maintained till 24 h. A similar observation was made for pH 6.4 and 7.0, where the inhibition of 50–65% was maintained from 4 to 24 h. A notable observation is that the potentiation activity of TAP was enhanced at acidic of pH 5.8 as well as slightly alkaline (pH 7.6). The time scale mapping reveals that at pH 5.8 and 7.6, the antimicrobial activity is enhanced from the initial time point till 24 h. A time-dependent gradual increase in the growth inhibition was observed with >75% inhibition at pH 5.8 and 7.6.

### Gene Expression Analysis

**Figure 3:**
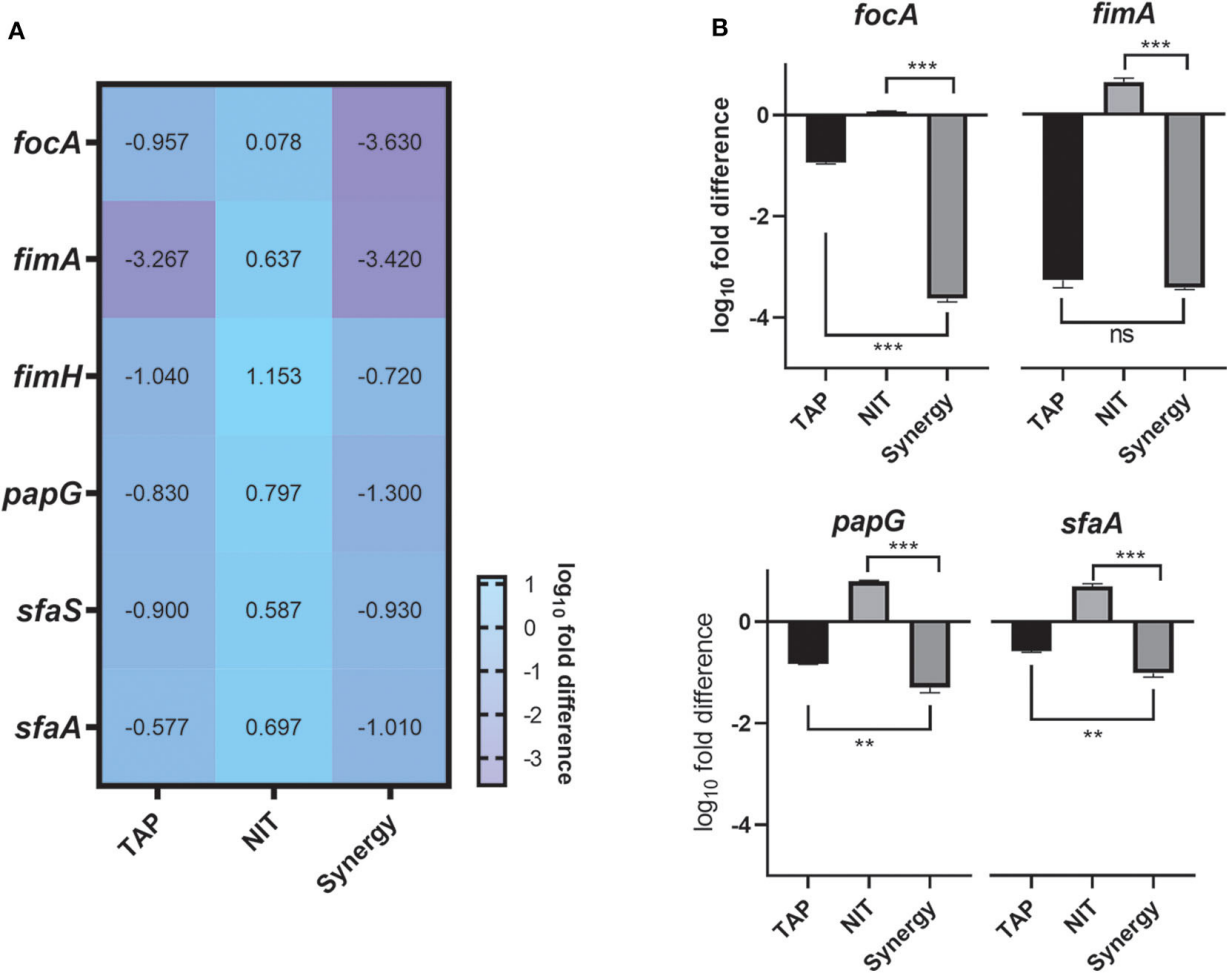
Gene expression Studies. **(A)** Heatmap of the adhesion genes downregulated by the combination. **(B)** Tableau Graph that represents the fimbriae systems considered. The graph shows that the synergy treatment significantly downregulated the adhesin genes as compared to the NIT treatment. The log_10_fold difference of the different genes upon different treatments is depicted. All the assays were done in triplicates on different occasions. The combination treatment and TAP downregulated adhesins considered. Student unpaired *t*-test was used for the significance analysis. *p* < 0.05 was considered significant. **p ≤ 0.001 and ***p ≤ 0.0001.

The above studies established the anti-biofilm and potentiating activity of TAP. In order to understand the biofilm inhibitory role of TAP and the synergy combination, gene expression analysis was carried out at pH 5.8 (Figure 3). The genes that control bacterial adherence was considered as they are mainly responsible for the bacteria to adhere to the urinary tract. The combination treatment showed a ~4 log_10_-fold reduction in the expression of *focA*, which encodes for the significant fimbrin subunit and belongs to the F1C fimbriae family. This was followed by P-fimbriae component, *papG* with a ~3.5 log_10_-fold reduction. The type I fimbrial adhesion system, *fimA* and *fimH* were also downregulated with ~2 log_10_-fold reduction. When compared to the other fimbriae systems considered, the S fimbriae (*sfaA* and *sfaS*) did not have a significant downregulation. There was only ~1 log_10_-fold reduction observed. A noteworthy observation is that the adhesion systems considered were slightly upregulated with only nitrofurantoin treatment. There was a proportionate downregulation of the fimbriae genes found with the TAP treatment. The downregulation of the adhesins was enhanced in the synergy treatment as compared to the individual treatments.

## Discussion

Urinary Tract Infections are placed at fourth rank among the infections associated with healthcare (Tenney et al., 2018). It is one of the most common diseases in the community as well as hospital settings. The primary uropathogen responsible for both complicated and uncomplicated UTI is Uropathogenic *E. coli* (UPEC) (Flores-Mireles et al., 2015). With the arsenal of virulence factors, UPEC invades the host system and causes infection (Terlizzi et al., 2017). Different classes of antibiotics are administered to contain the infection (Terlizzi et al., 2017). But the antibiotic treatments are failing due to a sharp increase in antibiotic resistance, and there is a need for alternative therapies. Biofilm is considered to be the major virulence factor of UPEC which promotes adherence to the host and thereby colonizing the host leading to a severe case of infection (Flores-Mireles et al., 2015).

The current study reports the antibiotic susceptibility pattern of the clinical isolates of UPEC and screening of Multidrug-resistant (MDR) strains among the clinical isolates. Multidrug resistance is defined when the bacterial strain is resistant to at least one agent in three and more than three antimicrobial categories (Magiorakos et al., 2012). According to this definition, the current study classifies all the 13 clinical isolates of UPEC are multidrug-resistant. The strains are resistant to the fluoroquinolones, second and third-generation cephalosporins, folate pathway antagonist, and quinolones. All the strains are nalidixic-resistant.

The clinical isolates are then analyzed for their biofilm-forming capacity. Biofilm forming capacity is evaluated both quantitatively (crystal violet method) as well as qualitatively (congo red agar method). The results of both methods correlated well with each other. The obtained strains are classified into strong, moderate and weak biofilm producers by their specific biofilm formation index value. Among the strong biofilm producers, three isolates were MDR (QSLUPEC3, QSLUPEC5, and QSLUPEC7). The strong biofilm production correlates significantly with the resistance to multiple class of antibiotics such as cephalosporins (second and third generation), quinolones, aminopenicillin, and fluoroquinolone. A strong biofilm producer being multidrug-resistant strain is well-documented previously (Murugan et al., 2011; Ponnusamy et al., 2012; Mittal et al., 2015). Biofilm gives multiple advantages to the pathogen for its survival, such as protection against host defense mechanism and antibiotic tolerance and resistance. Also, it is documented that the crucial event of the adhesion of the bacterial cells to uroepithelial cells is regulated through biofilm-forming factors, adhesins (Wu et al., 1996). Previous reports have proven that the biofilm formation is correlated to the increased hemolysin production and type 1 fimbriae expression, which are essential virulence factors (Soto et al., 2007). Thus, screening of the multidrug-resistant, strong biofilm producer as an ideal candidate to check the drug action becomes an eventuality. Hence for the further assays, the clinical isolate QSLUPEC7 was chosen, since it was a strong biofilm producing MDR strain.

Plant polyphenols are secondary metabolites that are mainly produced as plant defensive mechanisms (Daglia, 2012). They have various activities such as antioxidants, anti-allergic, anti-inflammatory, anti-cancer, anti-hypertensive, and antimicrobial agents (Daglia, 2012). It has also been established as an anti-biofilm agent, and researchers showed that biofilm mechanisms such as quorum sensing and other regulatory systems had been downregulated by the plant polyphenols without any effect on their growth (Slobodníková et al., 2016). A-type procyanidins from cranberry have been extensively studied for their anti-adhesive properties, especially against UPEC (Foo et al., 2000; Rane et al., 2014). They possess double interflavanyl linkages which confer the antiadhesive properties to these phytomolecules. It was shown that type-A procyanidin trimers attach to the P-fimbriae of UPEC preventing the adhesion to the uroepithelial cells. The anti-adherence activity for the type A proanthocyanidin trimer from cranberry was seen at a concentration of 2.4 mg/ mL (Foo et al., 2000). In the current study, the anti-biofilm activity of the type-A procyanidin pentamer from cinnamon is shown at a 15 μg/mL (MBIC_50_). The presence of the four interflavanyl linkages present in the pentamer may be attributed to the anti-biofilm activity of the TAP at a lower concentration.

Proanthocyanidins from cranberries were proven to have synergistic activity with antibiotics against both Gram-positive and Gram-negative organisms. Against *Staphylococcus aureus*, proanthocyanidins were shown to have synergy with the β-lactam antibiotics, as it acts on peptidoglycan synthesis (Diarra et al., 2013). A recent reported that the synergistic action of cranberry proanthocyanidins against Gram-negative organisms was through the repression of the intrinsic resistance mechanisms (Maisuria et al., 2019). In a study against *Pseudomonas aeruginosa*, it was proved that the potentiating activity of the proanthocyanidins due to its iron-chelating property and anti-biofilm property (Ulrey et al., 2014). Thus, biofilm inhibition can potentiate the action of the existing antibiotic (Vasudevan et al., 2018). The present study extends the antibiotic potentiating activity of the cinnamon derived proanthocyanidin to nitrofurantoin. Since it was reported that pH plays a vital role in the antibacterial activity of nitrofurantoin (Fransen et al., 2017), the synergy studies were conducted in a range of pH from 5.2 to 7.6. In order to establish synergy mathematically, two models were considered—Fractional Inhibitory Concentration Index and Bliss independence model (Kaur et al., 2016). There was at least 4-fold reduction in the MIC of NIT at all the pH considered. The maximum inhibitory activity of 80% was observed at pH 5.8, with a reduced concentration of NIT (8 μg/mL) in a time-dependent fashion. At pH 7.6, a similar result was obtained but at a higher concentration (16 μg/mL) but less than the CLSI guidelines of 32 μg/mL. There were conflicting results with respect to pH 5.2 where FIC index showed synergy, but Bliss score showed weak synergy. Thus, it is necessary to take into consideration more than one synergy model for the precise interpretation of the synergy activity.

The establishment of antibiofilm activity of TAP and the synergy with nitrofurantoin led us to unravel the mechanism of action of TAP and the combination. Adhesins are the portal of entry for UPEC to invade and persist in the dynamic host environment (Mulvey, 2002). They play an important role in the establishment of various virulence pathways, including biofilm formation (Behzadi, 2018). The antibiofilm activity of TAP can be attributed to the downregulation of the majority of the adhesins. Each of the adhesins has an essential and unique role in the pathogenesis process. The most downregulated adhesin is *focA* which is the primary fimbrin unit of F1C fimbriae unit. This is responsible for the ascending UTIs and has an affinity toward the different host cells, which include bladder and kidney epithelial cells (Mulvey, 2002). It should be noted that TAP did not have much effect on S-fimbriae genes which are homologous to F1C fimbriae. *papG* is closely associated with pyelonephritis, which was downregulated by TAP. This encodes for PapG protein which can adhere to erythrocytes and it was shown previously that the cranberry proanthocyanidin binds to P-fimbriae to exert anti-adherence property (Foo et al., 2000; Howell et al., 2005). The type I fimbriae system, *fimA* and *fimH*, is also downregulated. The type 1 fimbriae play a significant role in the bacterial adhesion (Bouckaert et al., 2006), and several studies were conducted to develop FimH inhibitors to evade UPEC pathogenesis (Han et al., 2010, 2012; Spaulding et al., 2017). It was shown that there is a cross-talk in the expression of adhesins regulation. The appearance of one adhesin suppresses the other. Thus, it is required to identify the compounds which can affect multiple adhesins. TAP alone and in combination with NIT was able to downregulate the major adhesins significantly that mediate UPEC attachment to host cells.

## Conclusions

A wide range of research is being done on the antimicrobial activity of the phytomolecules for the past few years as we are forced to identify alternative strategies to combat the antimicrobial resistance crisis. The ideal characteristics of these polyphenols selected for this study are their bioavailability, diverse structures, and non-toxic nature. This can help in developing them as excellent antimicrobial agents. Various studies also reveal the synergistic effects of Polyphenols and antibiotics. Through this study, the anti-biofilm effect and synergistic effect with the first-line antibiotic used for the uncomplicated UTIs, Nitrofurantoin was established. In the face of dynamic host condition, it is necessary to identify a cocktail of drugs which can evade the bacterial pathogenesis. The synergy studies revealed that the antibacterial activity was enhanced at a lower concentration of the nitrofurantoin at varying pH. Gene expression studies demonstrated the downregulatory effect on UPEC adhesins. Thus, the potentiating effect may be attributed to the biofilm inhibitory and downregulation of adhesins. Further validation studies are required to understand the target(s), the change in the gene expression of the adhesins at different pH and *in vivo* studies which are in process. This study reveals a promising lead to explore the combinatorial effect of Phyto-molecules and the currently used antibiotics.

## Data Availability Statement

All datasets presented in this study are included in the article/Supplementary Material.

## Author Contributions

All authors listed have made a substantial, direct and intellectual contribution to the work, and approved it for publication.

## Conflict of Interest

SB was employed by Indus Biotech Private Limited. The remaining authors declare that the research was conducted in the absence of any commercial or financial relationships that could be construed as a potential conflict of interest.
