# Supplementary material for: Reciprocal Cooperation of Type A Procyanidin and Nitrofurantoin Against Multi-Drug Resistant (MDR) UPEC: A pH-Dependent Study
Source: Front Cell Infect Microbiol. 2020 Aug 11;10:421. doi: 10.3389/fcimb.2020.00421 (PMC7431559; doi:10.3389/fcimb.2020.00421)
Supplement: Supplementary file 1 [file Data_Sheet_1.DOCX]

**Supplementary File**

**Reciprocal Cooperation of Type A Procyanidin and Nitrofurantoin against Multi-Drug Resistant (MDR) UPEC: A pH-dependent Study**

**Authors:** Sahana Vasudevan^1^, Gopalakrishnan Thamil Selvan^1^, Sunil Bhaskaran^3^, Natarajan Hari^2^ and Adline Princy Solomon^1*^

***Table S1: Primers used in this study***

| ***Gene*** | ***Primer Code*** | ***Sequence*** | ***Tm*** | ***Reference*** |
| --- | --- | --- | --- | --- |
| 16srRNA | 16R | 5’- TCTCGCGAGGTCGCTTCT-3’ | 58.7 | (Amalaradjou et al., 2011) |
|  | 16F | 5’- CCAGGGCTACACACGTGCTA-3’ | 59.3 |  |
| *sfaS* | S2F | 5’- TCTCACCGGATGCCAGAATAT-3’ | 55.7 |  |
|  | S2R | 5’ – GCCCATCCGCAGTACCACTA-3’ | 59.2 |  |
| *sfaA* | S1F | 5’- CCGTTCCAGGCTCGCTATATT-3’ | 56.9 |  |
|  | S1R | 5’- CGGCGTTGGCTGTACCA - 3’ | 58.5 |  |
| *fimH* | F2F | 5’- GATGCGGGCAACTCGATT-3’ | 55.8 |  |
|  | F2R | 5’ – CCCTGCGCGGGTGAA-3’ | 59.1 |  |
| *papG* | P1F | 5’- GGGAGGGAATGTGGTGATTACTC-3’ | 57.1 |  |
|  | P1R | 5’- CGGGCGCCACGAAGT-3’ | 59.3 |  |
| *focA* | FoR | 5’ – CGTCGGCGTTGGCAATA-3’ | 56 |  |
|  | FoF | 5’- CAGGCGGTTTACTACGCAACT-3’ | 57.6 |  |
| *fimA* | F1R | 5’-ACGCAGTCCCTGTTTTATCCA-3’ | 56.5 |  |
|  | F1F | 5’-TGCGGGTAGCGCAACAA-3’ | 58.1 |  |

**Table S2: Antibiogram of the UPEC clinical isolates. Co-Trimoxazole (COT-25), Trimethoprim (TMP-5), Cefuroxime (CXM-30) ,Cefotaxime (CTX-30), Nalidixic acid (NAL-30), Norflaxacin (NOR-10), Ciprofloxacin (CIP-5), Ampicillin (AMP-10) and Streptomycin (STS-10).** ^a^Zone of Inhibition in mm (Mean ± Standard Deviation); ^b^No Zone of inhibition

| **UPEC Clinical Isolates** | **Folate pathway antagonist** | | **Cephalosporin** | | **Quinolone** | **Fluoroquinolone** | | | **Aminopenicillin** | **Aminoglycoside** |
| --- | --- | --- | --- | --- | --- | --- | --- | --- | --- | --- |
| Clinical Break Points for Resistance | **COT**  $\boldsymbol{\leq}$ **10 mm** | **TMP**  $\boldsymbol{\leq}$ **10 mm** | **CXM**  $\boldsymbol{\leq}$ **14 mm** | **CTX**  $\boldsymbol{\leq}$ **22 mm** | **NAL**  $\boldsymbol{\leq}$ **13 mm** | | **NOR**  $\boldsymbol{\leq}$ **12 mm** | **CIP**  $\boldsymbol{\leq}$ **21 mm** | **AMP**  $\boldsymbol{\leq}$ **13 mm** | **STS**  $\boldsymbol{\leq}$ **11 mm** |
| QSLUPEC1 | **^a^**33 ± 0.62 | 27 ± 0.73 | 18 ± 0.65 | 31 ± 0.42 | - | | 26 ± 0.36 | - | - | 21 ± 0.31 |
| QSLUPEC2 | 20 ± 0.46 | 29 ± 0.28 | - | - | - | | 30 ± 0.41 | - | - | 13 ± 0.22 |
| QSLUPEC3 | 17 ± 0.61 | ^b^- | - | 32 ± 0.65 | - | | 33 ± 0.23 | - | - | - |
| QSLUPEC4 | 30 ± 0.81 | 28 ± 0.65 | 20 ± 0.38 | 32 ± 0.56 | - | | 26 ± 0.81 | - | - | 20 ± 0.81 |
| QSLUPEC5 | - | - | - | - | - | | - | - | - | 21 ± 0.76 |
| QSLUPEC6 | 25 ± 0.71 | 30 ± 0.61 | 23 ± 0.46 | 30 ± 0.63 | - | | 35 ± 0.66 | - | - | - |
| QSLUPEC7 | 27 ± 0.37 | 26 ± 0.46 | - | - | - | | - | - | - | - |
| QSLUPEC8 | - | - | - | - | - | | - | - | - | 21 ± 0.25 |
| QSLUPEC9 | 20 ± 0.4 | 26 ± 0.55 | 24 ± 0.25 | - | - | | - | - | - | 15 ± 0.91 |
| QSLUPEC10 | 22 ± 0.2 | 32 ± 0.43 | - | 35 ± 0.19 | - | | - | - | - | 16 ± 0.21 |
| QSLUPEC11 | - | 33 ± 0.81 | 19 ± 0.34 | 33 ± 0.28 | - | | - | - | - | 20 ± 0.36 |
| QSLUPEC12 | 17 ± 0.62 | 30 ± 0.56 | - | - | - | | 33 ± 0.33 | - | - | 21 ± 0.58 |
| QSLUPEC13 | - | - | 18 ± 0.67 | 33 ± 0.77 | - | | 34 ± 0.21 | - | - | 22 ± 0.71 |

**Table S3: Biofilm Forming Capacity of the clinical isolates: a) Crystal Violet Assay: Specific Biofilm Formation index (SBF) > 1.10 Strong Biofilm Producer; 0.7-1.09 Moderate Biofilm Producer; 0.35- 0.69 Weak Biofilm Producer b) Congo Red Agar (CRA) Assay: + Strong biofilm producer; - Weak Biofilm producer**

| **UPEC Clinical isolates** | **Specific biofilm formation index (SBF)** | **Interpretation** | **CRA** |
| --- | --- | --- | --- |
| QSLUPEC1 | 0.773 ± 0.05 | Moderate | + |
| QSLUPEC2 | 1.047 ± 0.03 | Moderate | - |
| QSLUPEC3 | 1.141 ± 0.085 | Strong | + |
| QSLUPEC4 | 1.842 ± 0.044 | Strong | + |
| QSLUPEC5 | 3.649 ±0.052 | Strong | + |
| QSLUPEC6 | 1.519 ± 0.036 | Strong | + |
| QSLUPEC7 | 2.339 ± 0.075 | Strong | + |
| QSLUPEC8 | 0.830 ± 0.024 | Moderate | + |
| QSLUPEC9 | 0.869 ± 0.058 | Moderate | + |
| QSLUPEC10 | 0.830 ± 0.069 | Moderate | + |
| QSLUPEC11 | 1.148 ± 0.013 | Strong | + |
| QSLUPEC12 | 0.625 ± 0.096 | Weak | - |
| QSLUPEC13 | 1.199 ± 0.055 | Strong | + |

***Figure S1****. The antibiotic resistant profile of Nitrofurantoin against QSLUPEC7.According to CLSI guidelines ≥ 64 µg/mL is the intermediate zone and ≥ 128 µg/mL is resistant.*


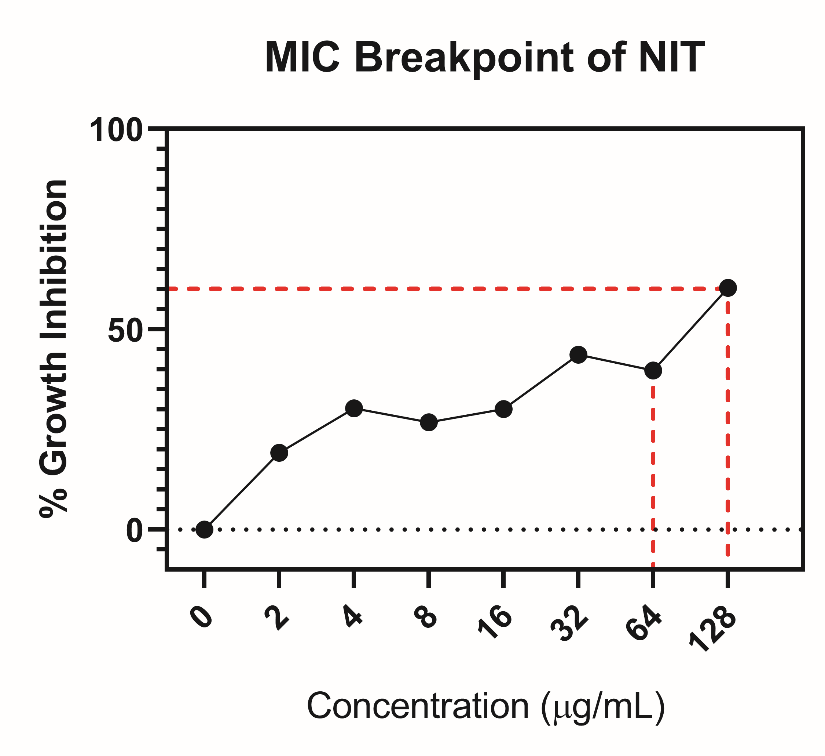


**References:**

Amalaradjou, M. A. R., Narayanan, A., and Venkitanarayanan, K. (2011). Trans-cinnamaldehyde decreases attachment and invasion of uropathogenic Escherichia coli in urinary tract epithelial cells by modulating virulence gene expression. *J. Urol.* 185, 1526–1531. doi:10.1016/j.juro.2010.11.078.

CLSI. Performance Standards for Antimicrobial Susceptibility Testing. 30th ed. CLSI supplement M100. Wayne, PA: Clinical and Laboratory Standards Institute; 2020.
